# Disruptive Behavior at Hospitals and Factors Associated to Safer Care: A Systematic Review

**DOI:** 10.3390/healthcare10010019

**Published:** 2021-12-23

**Authors:** Pedro Moreno-Leal, César Leal-Costa, José Luis Díaz-Agea, Ismael Jiménez-Ruiz, Antonio Jesús Ramos-Morcillo, María Ruzafa-Martínez, Adriana Catarina De Souza Oliveira

**Affiliations:** 1 Faculty of Nursing, Universidad Católica de Murcia, 30107 Murcia, Spain; pedromorenoleal@gmail.com (P.M.-L.); acatarina@ucam.edu (A.C.D.S.O.); 2 Faculty of Nursing, Universidad de Murcia, 30120 Murcia, Spain; Ismael.jimenez@um.es (I.J.-R.); ajramos@um.es (A.J.R.-M.); maruzafa@um.es (M.R.-M.)

**Keywords:** disruptive behavior, patient safety, doctor-nurse relationships, adverse effects, systematic review

## Abstract

Disruptive behavior creates a dysfunctional culture that has a negative impact on work relations and influences the quality of care and safety of the patient. The objective of the present work is to provide the best methodological quality scientific evidence available on disruptive behavior at a hospital, the aspect associated with the safety of the patient, and its impact on quality of care. For this, we included studies that addressed the prevalence of disruptive behaviors observed in the area of hospital health and its professionals. The selection, eligibility, data extraction and evaluation of the risk of bias stages were conducted by two researchers, and any discrepancies were solved by a third researcher. The data presented show that disruptive behaviors are frequently observed in the daily life of health professionals, and compromise the quality of care, the safety of the patient, and can lead to adverse effects. The results presented indicate that the appearance of disruptive behaviors compromises the quality of care, the safety of the patient, and the appearance of adverse effects, and can also affect the physical and mental health of the health professionals. PROSPERO registration number: CRD42021248798.

## 1. Introduction

Although no clear consensus exists about the definition of disruptive behavior, authors agree that it creates a dysfunctional culture that has a negative impact on work relations, making difficult interpersonal communication and influencing the quality of care and safety of the patient [1,2,3,4,5,6,7,8].

According to Meneses et al. [8], the main attributes of disruptive behavior are lack of civility and psychological violence, together with consequences such as moral and psychiatric suffering and broken communication. Important information is not shared, resulting in submission and little autonomy. Health centers cannot protect or ignore intimidating or perturbing behaviors, in order to not promote the insecurity of the patients in work contexts that are not healthy for the entire team [9].

In 2008, the Joint Commission International (JCAHO) considered disruptive behavior as threatening, inappropriate behavior, which has a deconstructive effect on the culture of safety, and for this reason, considered it as a Sentinel Event Alert 40, indicating the importance of preventing, managing, and controlling these behaviors among health professionals, including the managing board of the organization. It proposes eleven institutional strategies, highlighting effective and affective leadership and communication. It states that it is the responsibility of the institution to control a hostile environment and provide a proactive culture for safer care, aside from underlining the increase in healthcare costs [10].

Disruptive behaviors are perceived by health professionals as predictors of adverse events (53%), negative impacts with damage to health care (73%), and a contributor to mortality (25%) [11]. In this scenario, we also find medication mistakes linked to the bad relationship between the doctor and nurse. More specifically, it is attributed to the bad communication of verbal orders from the doctor related to the administration of medicines, when doctors become irritated when nurses do not follow their verbal orders until the content of these orders is not clarified [12]. This reminds us of the importance of the indicator “the spoken repetition of the verbal orders (a standardized manner for ensuring understanding)…” as part of the goal “Facilitate an adequate transfer of information and clear communication” proposed by the National Quality Forum of the United States [13] within the framework of “Safe Practices for Better Health Care”, whose objective is to implement and promote indicators of good practices to improve the level of safety of the patient.

Hicks et al. [14] found a variety of disruptive behaviors which entailed the worst clinical practice results, significantly harming the culture of safety. According to Shen et al. [15], one of the main factors which made it difficult to address disruptive behavior in clinical practice was perhaps the “culture of silence”, turning it into a complex process for health workers and institutions, and influencing the safety of the patient.

Therefore, the objective of the present systematic review is to provide the scientific evidence with the best methodological quality, on disruptive behavior at a hospital, the aspects related to the associated safety of the patient, and its impact on the quality of care.

## 2. Materials and Methods

### 2.1. Study Design

This systematic review study was conducted based on the updated version of the Preferred Reporting Items for Systematic Reviews and Meta-Analyses (PRISMA, 2020) [16,17]. This study was registered in the International Prospective Register of Systematic Reviews platform (PROSPERO), on 27 May 2021 (Registration number: CRD42021248798).

### 2.2. Selection Criteria

#### Eligibility Criteria

The eligibility criteria adopted in the review are related to the research questions: Which are the most frequent disruptive behaviors in the hospital environment and what are their impacts on the safety of the patient? Which are the triggers of these disruptive behaviors perceived by the health professionals?

Therefore, the inclusion criteria were chosen to start with a condition, context, and population (CoCoPop) [18]. Thus, the following studies were selected: those which addressed the prevalence of disruptive behaviors (Co); developed in the area of hospital health (Co); and which evaluated the disruptive behavior of health professionals (Health professionals who provided direct or indirect care, of any gender or race, with at least 3 months of service at any hospital services or unit), and their consequences on the safety of the patient (Pop). We chose to work with studies published between 2014 and 2021, as we observed that studies about the subject were widely investigated in the last 5 years.

As result, studies with non-resident students and studies conducted strictly with the administration of hospital institutions were excluded.

### 2.3. Sources of Information and Bibliographic Search

Initially, various search strategies were tried in each database. Based on the greater sensitivity and specificity, a standardized search strategy was defined, after which two researchers performed the search independently. The search terms and descriptors (social behavior, disruptive behavior, patient safety, and health professionals, were combined with the Boolean operators AND / OR. The search strategies adopted were applied to the databases selected (PubMed, Web of Science, ScienceDirect, PsycINFO and CINAHL).

After the search was conducted in the databases, the article files were collected with the application Rayyan, with which an initial verification was performed to detect the presence of duplicate articles.

During the selection phase, the titles and abstracts of the articles were read to evaluate their compliance with the eligibility criteria. Then, the complete text of the articles selected was read. Additionally, the references from these articles were examined to detect studies that could be potentially relevant. At all the stages, two reviewers were responsible for the reading of the articles, and when a divergence was observed, a third reviewer was consulted.

### 2.4. Data Extraction

The general information and methodology utilized were collected from each study: title, first author, year of publication, country, the objective of the study, type of disruptive behavior and its frequency, impact on the safety of the patient, and quality of attention, and lastly, type of measuring tools, data analysis, and main results.

### 2.5. Evaluation of Risk of Bias

The JBI scale (Joanna Bridges Institute) [19] was utilized to evaluate the methodological quality (risk of bias) of the cohort studies (longitudinal) and cross-sectional. The evaluation process was performed by two independent researchers, and any doubt or disagreement was resolved with the help of a third researcher.

### 2.6. Data Analysis and Synthesis

The general information and the methods applied in the study were extracted. We collected the name of the authors and year of publication, the size of the sample, the measurement instruments utilized in the evaluation of disruptive behaviors, and the main results presented in each study. There was high heterogeneity in the characteristics and size of the samples, in the results, and in the measurement instruments. For these reasons, quantitative synthesis of the data was not possible.

## 3. Results

**Figure 1:**
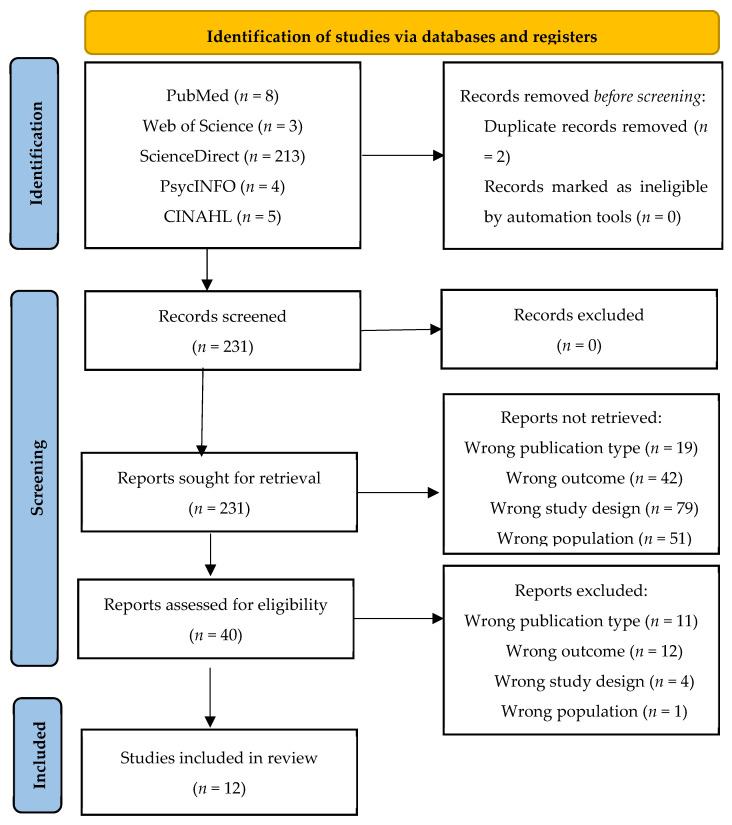
This figure shows the flow diagram utilized for the review, based on the PRISMA model (2020).

The search in the different databases provided 233 articles, which were screened to evaluate their eligibility. Of these, two were eliminated because they were duplicates. After reading the titles and abstracts, and after reading the complete articles, 12 articles were selected for their systematic review [20,21,22,23,24,25,26,27,28,29,30,31] (Figure 1).

Of the 12 studies selected, five were conducted in the Unites States [20,23,26,28,29], 2 in Canada [22,30], two in Israel [27,31] and one each in other countries (China, Egypt and Iran) [21,24,25]. The studies included 22,176 health professionals (nurses, doctors, medicine students and technicians).

As for the measurement instruments, high heterogeneity was observed. Different types of scales and questionnaires, validated and adapted to each studied population, were applied. Additionally, these were provided printed or virtually [20,21,22,23,24,25,26,27,28,29,30,31]. Starting with the results described in Table 1, we found that disruptive behavior was frequently observed in the routines of the health professionals [20,21,22,23,24,25,26,27,28,29,30,31]. With this respect, it was notable that most of the studies on the prevalence of disruptive behaviors in the hospital context could reach or exceed 90% of the occurrence in the last few weeks, months, or years [21,24,25,28,30,31].

The consequences of these disruptive behaviors are documented by health professionals. It was observed that its occurrence can compromise the quality of care, patient safety, and lead to adverse effects [20,21,25,27]. Aside from these negative consequences, it is observed that these disruptive behaviors can lead to physical health (musculoskeletal) and mental (stress, emotional exhaustion, depression, dissatisfaction at work, and sleep disorders) problems of the health professionals in a hospital context [20,21,22,23,24,25,26,27,28,29,30,31].

In general, the high prevalence of disruptive behaviors and consequences on the quality and safety of the patient, as well as the physical and mental damage of the health professionals re-enforces the need to monitor these behaviors and actions oriented towards collaborative work [31].

With respect to the evaluation of the methodological quality, some weaknesses were found in the evaluation of the exposure, the definition of the criteria to standardize the measurement of the condition, the identification of confounding factors, and the strategies to face these variables (items 2, 3, 4, 5, and 6). However, some studies showed an excellent methodological quality [22,26,27].

## 4. Discussion

The results presented in this systematic review indicate that the appearance of disruptive behaviors can compromise the quality of care, the safety of the patient, and can lead to adverse effects. In the studies included, it was observed that these behaviors can affect the physical and mental health of the health professionals, independently of their profession and length of employment.

At present, the studies on this subject are mainly cross-sectional, through the use of a questionnaire, and are mainly focused on four broad dimensions that are associated with the safety of the patient and the quality of care, considering the possible adverse effects experienced during the process of care derived from the different disruptive behaviors. These dimensions are: Motives and prevalence of disruptive behavior in the health context where the individual factors, the environmental factors, the organizational factors, and the social factors, are relevant, with important correlations observed between abuse and gender, physical abuse and position, and physical abuse and level of education that have generated at least one observed disruptive behavior [30,32,33]; places and moments in which these disruptive behaviors are produced; in this dimension, we highlight the recording of disruptive behavior at emergency services, surgery rooms, and ICU, considered as high complexity due to the variability of the processes and level of care, linked with the physical and emotional workload [20,23]; the types and element characteristics of disruptive behavior: the studies underlined the aspects associated with “intimidation” and “hostility” related with workload and teamwork [26,34]; the strategies and protocols: in most cases, the studies indicated the implementation of a proactive culture by the health professionals in the development of competences in patient safety, team work, and the making of decisions, and by the health institutions, for providing a functional and structural organization for the development of this culture, to promote human qualities at work [8,28,35,36].

With respect to the social-labor variables, a study that analyzed the reports of the notifications observed more disruptive behaviors in the category “physicians” (81%) compared to 52% in the category “nursing”. It was observed that these behaviors result in stress (97%), job dissatisfaction and compromised patient safety (53%), quality of care (72%) and errors (70%) [25].

Another study addressed 2821 nurses, who indicated the existence of a relationship between disruptive behavior and gender. Women indicated that they suffered more verbal and physical abuse than men [28].

### Study Limitations

The main limitation of the study is related to the absence of clinical trial studies on disruptive behaviors associated with patient safety. Given the heterogeneity of the studies included in the systematic review, a meta-analysis was not possible. Therefore, the findings of this systematic review have led to an update in scientific knowledge in this area, and the results can be further utilized to facilitate decision-making and the implementation of new health policies.

## 5. Conclusions

The results presented in this systematic review indicate that the appearance of disruptive behaviors compromises the quality of care in the hospital setting. These disruptive behaviors (rudeness, violence in the workplace, feeling of threat, poor distribution of workload and refusal to work in a team) lead to negative consequences, such as the safety of the patient, the appearance of adverse effects, and can also affect the physical and mental health of the health professionals, independently of their profession and the length of employment. Most importantly, it is hoped that these data can be used to develop organizational policies to enhance the workplace and positive patient outcomes within a healthy work environment.

## Figures and Tables

**Table 1:** Characteristics of the studies included in the systematic review.

Author (Year)	Country	Participants	Measuring Instruments	Key Findings
Addison and Luparell (2014) [20]	United States	57 nurses (M = 54 and F = 31)	The questionnaire consists of 21 questions (Dr. Alan Rosenstein’s questionnaire)	Nurses perceived that disruptive behavior is linked to adverse events and may also have a negative impact on patient safety and satisfaction.
Elhoseny and Adel (2016) [21]	Egypt	120 Physicians (M = 89 and F = 31)	A self-administered questionnaire of 16 questions based on the ACPE and QuantiaMD Survey	98.3% of respondents reported that disruptive behavior affects patient care. The most frequent behavior was the refusal to cooperate with other providers (74.2%), with 35% of the interviewees.
Havaei; Astivia and McaPhe (2020) [22]	Canada	537 medical-surgical nurses (M = 26 and F = 506)	A series of four workplace violence items were adapted	Burnout, a psychological response to workplace violence, increased nurses’ reports of musculoskeletal injuries, anxiety disorders, and sleep disturbances.
Keller et al. (2018) [23]	United States	1208 nurses (M = 206 and 1102)	22 multi-item scales that were drawn from existing valid and reliable instruments	One individual (marital status) and three workplaces (setting, schedule, and role) characteristics, as well as one dispositional (negative affectivity), one contextual (organizational constraint), and two interpersonal (distributive justice and workgroup cohesion) factors, were significant predictors of RNs’ experiences of verbal abuse by RN colleagues.
Liut et al. (2021) [24]	China	1481 nurses (M = 116 and F = 1365)	The Workplace Psychological Violent Behaviors instrument	Chinese surgical nurses reported a high prevalence of WPV (92.1%), which is substantially higher than that seen in other departments in China.
Maddineshat et al. (2016) [25]	Iran	45 physicians e 110 nurses	A translated and modified 25-item questionnaire	81% of physicians and 52% of nurses exhibited disruptive behavior. It was observed that these behaviors result in stress (97%), job dissatisfaction and compromised patient safety (53%), quality of care (72%) and errors (70%).
Rehder et al. (2020) [26]	United States	7.293 health care workers (M = 1658 and F = 5.906)	Disruptive Behavior Scale (6 items)	Significant correlations of disruptive behaviors with a worse teamwork climate, safety climate, job satisfaction and management of perceptions were detected; a lower balance between work and personal life; increased emotional exhaustion; and increased depression.
Riskin et al. (2019) [27]	Israel	160 medical professionals	A Likert scale was utilized (1 = not much to 5 = extremely) with six questions	Exposure to rudeness is not only associated with increased state depletion and decreased information sharing among team members, but it may put patients’ safety at risk given its association with reduced compliance with infection control and medication protocols.
Small; Porterfield and Gordon (2015) [28]	United States	2821 nurses (M = 276 and F = 2512)	21 web-based questions were utilized to assess disruptive behaviors	Most participants reported that the work environment presented a high risk for the occurrence of at least one disruptive behavior every 6 months.
Stecker and Stecker (2014) [29]	United States	617 nurses (M = 75 and F = 542)	The Provider Conflict Questionnaire and the Perceived Stress Scale	The importance of workplace stress is emphasized by the fact that more than 78% of the respondents in our study felt that most of their stress came from work rather than home.
VillaFranca et al. (2019) [30]	Canada	7465 anesthesiologists, nurses, surgeons, senior medical students and technicians (M = 3836 and F = 3629)	14 examples of disruptive behaviors were measured in a Likert scale of 7 points	Nearly all respondents reported experiencing or witnessing multiple examples of disruptive behavior, with an average respondent observing between 12 and 61 events within the preceding year.
Warshawski (2015) [31]	Israel	262 nurses	A structured questionnaire	Nurses in this sample reported a high sense of professional threat maybe as a result of an ongoing lack of collaboration with other team members.

## Data Availability

The datasets used and/or analyzed during the current study are available from the corresponding author on reasonable request.

## Abbreviations

JBI	Joanna Bridges Institute
PRISMA-P	Preferred Reporting Items for Systematic Reviews and Meta-Analyses-Protocols
SR	systematic review
